# Speech Perception in Noise: A Narrative Review of Cognitive Contribution and Hearing-Aid Signal Processing

**DOI:** 10.3390/jintelligence14090229

**Published:** 2026-09-21

**Authors:** Daniele Monzani, Andrea Bianchino, Andrea Ciorba, Chiara Bianchini, Marianna Manuelli, Andrea Migliorelli, Silvia Palma

**Affiliations:** 1 ENT, Department of Surgical Sciences, Dentistry, Gynaecology and Paediatrics, University of Verona, Borgo Roma Hospital, 37134 Verona, Italy; daniele.monzani@univr.it; 2 ENT & Audiology Unit, Department of Neurosciences, University Hospital of Ferrara, 44121 Ferrara, Italy; andrea.bianchino@edu.unife.it (A.B.); andrea.ciorba@unife.it (A.C.); chiara.bianchini@unife.it (C.B.); marianna.manuelli@ospfe.it (M.M.);; 3 Audiology, Primary Care Department, AUSL of Modena, 41100 Modena, Italy

**Keywords:** working memory, noise, speech perception, cognitive hearing science, hearing aid, artificial intelligence

## Abstract

Background: Environmental noise is one of the most pervasive ecological health issues in modern urban settings, contributing to the onset of hearing damage but also to the development of annoyance, sleep disturbance, cardiovascular diseases, and impaired communication. For people with hearing impairment of any age, the main challenge posed by noisy environments is not merely detecting sound but understanding speech against a competing background—a task heavily related to cognition. Methods: Narrative review; the literature has been identified through searches of PubMed, Scopus, and Google Scholar, combining terms related to cognition, speech perception, speech in noise, hearing-aid signal processing, environmental health, and aging. Results: The role of cognitive abilities in speech perception in noise has been evaluated, with particular attention to how artificial intelligence supports the interplay between cognition and hearing-aid signal processing in adverse environments. Within the Ease of Language Understanding (ELU) model, this paper describes how degraded acoustic input shifts processing from rapid, implicit lexical access toward explicit working-memory-dependent reconstruction. The actual evidence indicates (i) that working memory, in particular, could predict aided speech recognition under adverse conditions, (ii) that aging dissociates these processes, and (iii) that longitudinal studies link hearing-aid use to slower cognitive decline. Conclusions: Speech perception in noise is a coordinated neurocognitive process rather than a purely auditory task. Working memory is a central cognitive contributor, with effects that become evident specifically under adverse conditions. In particular, fast-acting compression and frequency lowering may be counterproductive for listeners in case working memory or speech processing are reduced. The success of different algorithms applied to audio signal processing of hearing aid, designed to compensate for difficult comprehension of words and phrases in noisy scenarios is also strictly dependent on working memory, and artificial intelligence significantly contributes to this goal.

## 1. Introduction

Environmental noise is a major and growing environmental health concern worldwide. The World Health Organization identified the levels at which noise causes significant harm and synthesized evidence linking it to cardiovascular and metabolic effects, annoyance, sleep disturbance, cognitive impairment, and hearing impairment and tinnitus (World Health Organization, 2018). Beyond hearing damage and tinnitus, chronic noise exposure has broader health effects and disproportionately affects vulnerable groups (Basner et al., 2014; Münzel et al., 2021). Those with hearing impairment are among the most affected, as the core function of communication can be compromised. Hearing sounds and understanding a speech are not the same task; even listeners with mild hearing loss may struggle to perceive speech in noise because of limitations in central auditory and cognitive processing rather than audibility alone. Such central auditory processing difficulties have an identifiable neurobiological basis and may share neurochemical substrates with neurodevelopmental conditions, underscoring that speech understanding in noise is constrained by central as well as peripheral factors (Bianchino et al., 2026).

Over the past two decades, several researchers have focused on understanding how sensory and cognitive systems interact during speech communication. This evolution reflects the recognition that understanding spoken language—particularly in acoustically challenging environments—requires far more than intact peripheral sensitivity. The emergence of cognitive hearing science represents a convergence of audiology, psychology, neuroscience, and linguistics, motivated by consistent evidence that individual differences in cognitive ability influence speech-perception outcomes independently of pure-tone thresholds (Arlinger et al., 2009).

The clinical implication is substantial: listeners with near-identical audiograms can experience markedly different real-world communication outcomes, suggesting that the success of rehabilitation heavily depends on central auditory processing rather than peripheral audibility or technological sophistication alone. A recent narrative review showed that, to fully understand the relationship between hearing and cognitive impairment, not only peripheral but also central hearing needs to be considered, and that comorbidity between the two conditions could impact on hearing-aid outcomes negatively (Nixon et al., 2019). The relevance of this perspective extends beyond the concept of effective communication with others. A meta-analysis in 2016 suggests that hearing impairment is associated with cognitive problems. However, due to diversity within studies, small sample sizes, and bias due other health factors, this conclusion should be accepted with caution (Taljaard et al., 2016).

Our narrative review focuses on working memory as a central cognitive contributor to speech perception in noise and also examines the theoretical basis for cognition–audition interactions, the empirical evidence linking these cognitive capacities to aided and unaided speech recognition, the way they interact with specific hearing-aid signal-processing strategies, and their trajectories across the lifespan. It also evaluates the most recent technical solutions—in particular, deep learning and artificial intelligence approaches to active noise reduction in hearing devices. Accordingly, the Results have been organized into the following sections: the theoretical framework (Section 3.1); working memory and speech perception in noise (Section 3.2); the interaction of working memory with hearing-aid signal processing (Section 3.3); cognitive processing speed as a complementary predictor (Section 3.4); lifespan trajectories and the aging listener (Section 3.5); the mechanisms linking hearing loss and cognition (Section 3.6); hearing-aid intervention and cognitive outcomes (Section 3.7); and artificial-intelligence approaches to noise reduction (Section 3.8).

## 2. Materials and Methods

The present is a narrative review. The literature has been identified through searches of PubMed, Scopus, and Google Scholar, combining terms related to cognition (“working memory”, “processing speed”, “cognitive hearing science”), speech perception (“speech-in-noise”, “speech recognition”, “intelligibility”), hearing-aid signal processing (“wide-dynamic-range compression”, “noise reduction”, “deep learning”, “artificial intelligence”), environmental health (“environmental noise”, “noise pollution”, “cardiovascular”, “sleep disturbance”), and aging (“age-related hearing loss”, “cognitive decline”, “dementia”). Authoritative reports and guidelines, including those of the World Health Organization, were also consulted. The search covered publications from 2006 to 2026, with the included sources ranging from foundational work on cognition and hearing-aid compression to the most recent lifespan and interventional studies. Priority was given to peer-reviewed empirical studies, influential theoretical papers, and quantitative syntheses. Studies were included (i) when addressing the relationship between cognition—in particular, working memory or perceptual speed processing—and speech perception in noise or the interaction of these abilities with hearing-aid signal processing and (ii) when English-language.

Reference lists of key papers were screened for additional sources. Because this is a narrative synthesis intended to integrate evidence across disciplines rather than to estimate a pooled effect, no formal quality-appraisal protocol or quantitative meta-analytic procedure was applied; the selection therefore reflects an interpretive rather than exhaustive sampling of the literature, and this is acknowledged as a limitation. The principal studies informing this review have been summarized in Table 1. The focus is on several issues such as (i) the theoretical framework that accounts for cognition–audition interactions; (ii) the links between working memory, perceptual speed processing, and speech perception in noise; (iii) the interaction between working memory and hearing-aid signal processing; (iv) the trajectories across the lifespan; (v) the connections among hearing loss and cognition; and (vi) the artificial-intelligence approaches to active noise reduction techniques of hearing aids.

## 3. Results

### 3.1. Theoretical Framework: The Ease of Language Understanding Model

The Ease of Language Understanding (ELU) model provides the principal theoretical framework for cognition–audition interactions (Rönnberg et al., 2013). The model proposes that successful language understanding depends on a match between incoming acoustic input through the rapid, automatic, multimodal binding of phonology and phonological representations stored in long-term memory (Rönnberg et al., 2022). When this match occurs readily, as in quiet with clear speech, lexical access proceeds rapidly and implicitly, consuming minimal working-memory resources. When the acoustic signal is degraded (hearing loss, background noise, or distortions introduced by signal processing), a phonological mismatch can occur. This mismatch triggers explicit, effortful working-memory engagement to reconstruct meaning through interaction with semantic and episodic long-term memory (Rönnberg et al., 2021). Thus, the model explains why cognitive demand increases systematically with signal degradation and why individual differences in working-memory capacity predict outcomes specifically under adverse listening conditions. A structural-equation analysis of hearing-aid users has supported this architecture, indicating that phonological processing predicts speech-recognition thresholds across listening conditions, that working memory becomes influential under adverse conditions, and that processing speed mediates relationships among the model’s parameters (Homman et al., 2023).

### 3.2. Working Memory and Speech Perception in Noise

Working memory is particularly important because it supports many higher-level cognitive skills and is a significant contributor to general cognitive abilities. However, working memory and general cognition are conceptually distinct, since the latter encompasses several abilities, including attention, processing speed, and executive functioning (Dryden et al., 2017).

#### 3.2.1. Current Theoretical Considerations on Working Memory

Working memory refers to the brain system that temporarily holds and manipulates information in the service of ongoing cognitive tasks, as distinct from the passive short-term storage of material (A. D. Baddeley & Hitch, 1974). The dominant account remains the multicomponent model of working memory (A. Baddeley, 2012; Hitch et al., 2025), which distinguishes a limited-capacity attentional controller—the central executive—from two domain-specific storage subsystems: the phonological loop, which maintains speech-based and acoustic information through rehearsal, and the visuospatial sketchpad, which holds visual and spatial material. A fourth component, the episodic buffer, was later added to explain how information from these subsystems and from long-term memory is bound into integrated, multimodal episodes (A. Baddeley, 2000). Fifty years after the model was first proposed, its core architecture has proved durable: recent appraisals retain the original components while assigning the episodic buffer a more central role, as a hub interfacing working memory with long-term memory and the focus of attention (A. Baddeley, 2012; Hitch et al., 2025). A complementary tradition emphasizes attention rather than dedicated stores. In Cowan’s embedded-processes model, working memory is not a separate structure but the subset of long-term memory currently in a heightened state of activation, with a focus of attention holding only about three to five meaningful items, or chunks, at once in young adults (Cowan, 2010). Contemporary syntheses converge on the view that working memory and attention are intimately linked and that capacity limits reflect the control of attention against interference at least as much as storage, per se (Oberauer, 2019). Indeed, current scholarship increasingly treats working memory not as a single fixed structure but as a set of partly overlapping mechanisms—storage, attentional control, and links to long-term knowledge—operating together (Logie et al., 2021). This characterization maps naturally onto listening in noise, where the target talker must be held and continuously updated while competing sounds are suppressed. The phonological loop is especially relevant to speech perception, being the component most directly engaged when degraded or unfamiliar acoustic input must be held and manipulated to recover meaning. This is why measures such as the reading-span test—which index the combined storage and processing capacity of verbal working memory—predict speech-in-noise outcomes (Foo et al., 2007). It is this active, capacity-limited, and attention-dependent system, rather than passive storage, that the Ease of Language Understanding model places at the center of effortful listening when the incoming signal fails to match stored phonological representations (Rönnberg et al., 2013, 2021).

#### 3.2.2. Working Memory and Adverse Listening Conditions

Working memory can be conceived as a limited-capacity system that is recruited when an input signal cannot be readily matched to a stored representation (Souza et al., 2015). Because such mismatches occur more frequently when the signal is degraded, working memory is expected to contribute to speech recognition most when listening conditions are adverse rather than favorable. This conditional relationship is the defining empirical signature of working memory in speech perception.

Evidence that the cognition–speech link is domain-specific comes from a Pilot Study in which 250 community-dwelling older adults (mean age 77 years) completed a clinical speech-in-noise measure alongside a neurocognitive battery (Mamo et al., 2019). After adjustment for hearing thresholds and demographic factors, each 1-dB increment in speech-in-noise difficulty (signal-to-noise-ratio loss) was associated with significantly lower standardized scores for memory (β = −0.80), language (β = −0.92), processing speed/executive function (β = −0.65), and a global composite (β = −1.11). In sensitivity analyses excluding participants with moderate or severe hearing loss, memory remained the only domain consistently associated with speech-in-noise performance, while the language and processing-speed associations attenuated. This pattern is consistent with the ELU prediction that episodic long-term memory and its interface with working memory represent the most robust cognitive linkage to speech perception when hearing function is not severely compromised (Mamo et al., 2019).

Auditory working-memory tasks may be particularly relevant to speech perception as they involve the encoding, preservation, and management of information presented in the same sensory modality. However, particularly in individuals with hearing loss, performance on such tasks may be also influenced by the input quality and audibility. Several tools have been designed and are described in the literature to assess “auditory” working memory, such as the Listening Span (LS/LWMS) task or the Sentence-final Word Identification and Recall (SWIR) test (Besser et al., 2013; Ng et al., 2013). It is well known that speech-perception outcomes obtained using syllables and isolated words and sentences are not equivalent, as these materials differ substantially in their linguistic complexity, contextual information, and cognitive demands. Sentence-based tasks in particular provide syntactic, semantic, and contextual cues that can facilitate the reconstruction of degraded speech (Souza et al., 2015).

### 3.3. Working Memory and Hearing-Aid Signal Processing

Before considering the interaction between hearing-aid signal processing and working memory, it is useful to summarize the general effects of these processing schemes on speech perception. WDRC is intended to restore audibility across the reduced dynamic range associated with hearing loss; however, its effects on speech perception in adverse conditions depend on several factors, including compression speed and ratio, number of channels, and the characteristics of the competing noise. In such conditions, the amplification of noise can impact the effective signal-to-noise ratio and the acoustic cues supporting speech understanding. Similarly, noise reduction algorithms do not always improve speech intelligibility, as their benefit is also related to the listening environment and to the signal acoustic features (Souza et al., 2015).

#### 3.3.1. Wide-Dynamic-Range Compression

The relationship between working-memory capacity and hearing-aid benefit depends on the specific signal-processing strategy employed, contradicting any simple expectation that greater cognitive ability always yields greater benefit from any amplification scheme (Souza et al., 2015). In a frequently cited study of 32 experienced hearing-instrument users, the authors examined aided speech recognition when participants were fitted with new, unfamiliar compression-release settings (Foo et al., 2007). Working-memory capacity, measured with the reading-span test, accounted for a substantial proportion of the between-listener variance in speech-reception thresholds—on the order of 40%—when the processing differed from listeners’ habitual settings, consistent with the ELU prediction that an unfamiliar, mismatching signal places heavier demands on explicit working-memory resources.

The literature further suggests that compression speed modulates this dependence. When slow-acting compression is applied, aided speech recognition tends to be governed primarily by age and degree of hearing loss rather than by working-memory capacity, whereas fast-acting compression—which produces greater moment-to-moment modification of the temporal envelope—engages working memory more heavily (Souza et al., 2015). The cognitive-demand profile also appears to shift with the linguistic predictability of the speech material: when ample context supports top-down prediction, the working-memory bottleneck is relaxed and processing speed becomes more limiting, whereas low-context material that requires genuine phonological decoding makes working-memory limitations more apparent (Souza et al., 2015). Recent Studies on Adaptive WDRC (AWDRC)—which dynamically adjusts time constants based on input changes—demonstrate significant improvements in signal-to-noise ratio (SNR), user comfort, and speech clarity in background noise. 

Comparable dependencies extend to other signal-altering strategies. Frequency compression, which lowers high-frequency energy into regions of better residual hearing, likewise distorts the signal and interacts with both age and cognition. In a study of 26 adults aged 62–92 years with hearing loss, working memory was a significant determinant of the intelligibility of frequency-compressed sentences in babble noise: at maximum signal modification, working memory accounted for roughly 29% of the variance in intelligibility once age and hearing loss were controlled, and working memory, age, and hearing loss together explained almost half (about 48%) of the between-listener variability (Arehart et al., 2013). This finding crystallizes the central message for age-related hearing loss: presbycusis, reduced working-memory capacity, and processing-induced distortion compound one another, so that the older listeners who are typically offered the most aggressive processing are often those least able to absorb its cognitive cost.

#### 3.3.2. Digital Noise Reduction

Digital noise reduction (NR) algorithms interact with working memory in ways that depend on signal-to-noise ratio, algorithm type, and listener features and that diverge somewhat from compression effects. Syntheses of executive-function and intelligibility studies indicate that larger working-memory capacity is generally associated with better performance under noise reduction, but that the interaction is conditional—for example, listeners with larger capacity have shown performance changes under strong (but not moderate) noise reduction that listeners with smaller capacity did not (Souza et al., 2015). In studies of binary-mask noise reduction with older hearing-aid users, those with larger working-memory capacity recalled more words, suggesting that effective suppression of background noise can free resources for downstream processing in higher-capacity listeners (Yumba, 2017). The relationship nonetheless remains inconsistent across studies, plausibly reflecting differences in algorithms, implementation, and adaptation period. Table 2 summarizes how the main processing strategies interact with cognitive demand.

#### 3.3.3. Directional Processing and Frequency Lowering

Directional (DIR) processing may also influence the cognitive demands associated with speech understanding in noise. By improving the signal-to-noise ratio and reducing interference from challenging sounds, directional processing may reduce the dependence on working-memory resources during speech processing (Desjardins, 2016).

Similarly, working memory could also be relevant in the case of frequency lowering (FL), as it can support the maintenance and integration of incomplete or unfamiliar speech information with linguistic knowledge. It is also likely that other factors—such as the degree of hearing loss, the degree of frequency lowering, and the listening conditions—may further influence this process (Simpson, 2009; Arehart et al., 2013).

### 3.4. Cognitive Processing Speed as a Complementary Predictor

Although working memory has historically dominated cognitive hearing science, cognitive processing speed has emerged as a particularly robust predictor of aided speech intelligibility. In a study of 194 hearing-aid users (aged 33–80 years) with bilateral mild-to-moderate sensorineural hearing loss, Yumba (2017) manipulated hearing-aid processing (linear amplification with and without noise reduction and fast-acting compression) and background noise while assessing processing speed with a lexical-decision task and working memory with a semantic word-pair span test, aiding recognition with Hagerman sentences. Cognitive processing speed was the better predictor of speech intelligibility in noise regardless of the signal-processing algorithm used, whereas working-memory capacity showed a weaker and more variable relationship—the strongest processing-speed associations appearing for noise reduction and fast-compression outcomes in steady-state noise.

These findings do not displace working memory so much as complement it. Because processing speed appears comparatively stable across algorithms and stimulus types, it may generalize more readily across heterogeneous hearing-impaired populations, while working-memory effects are more sharply conditional on the degree and type of signal degradation. Both constructs are, moreover, mechanistically linked within the ELU framework, where the speed of phonological and lexical processing constrains how efficiently working memory can be deployed to resolve a mismatch (Homman et al., 2023).

### 3.5. Lifespan Trajectories and the Aging Listener

Sensory and cognitive contributions to speech-in-noise perception follow different developmental timetables, and their divergence helps explain why older listeners are especially vulnerable. In a lifespan study of 357 individuals aged 7–90 years, speech reception thresholds in noise followed a U-shaped trajectory that peaked in the late thirties; structural-equation modeling indicated that hearing sensitivity and cognitive factors jointly mediated age effects, explaining about 61% of the variance (Bugannim et al., 2025). Notably, peripheral sensitivity began to deteriorate from around age 17, whereas cognitive measures including processing speed did not begin to decline until roughly ages 32–38, coinciding with the inflection point of speech-in-noise performance. This staggered pattern creates a compounding disadvantage in later life: age-related slowing of central processing combines with peripheral signal degradation to challenge real-time speech understanding, with speech-in-noise performance declining most steeply after the sixth decade. It is likely that several tools could be useful to assess these features: (i) behaviorally, through cognitive processing-speed tasks (such as lexical-decision or reaction-time measures) and tests of central auditory temporal processing (e.g., gap detection and temporal-modulation sensitivity); and (ii) electrophysiologically, through cortical auditory evoked potentials (Pichora-Fuller & Singh, 2006). The same interaction may underlie observations that older listeners respond differently from younger listeners to particular compression and processing characteristics, since reduced processing speed narrows the temporal window within which a degraded or rapidly modified signal can be resolved. Clinically, this convergence matters for amplification: because presbycusis tends to co-occur with reduced working-memory capacity, the same older listeners are often the most susceptible to the distortion introduced by complex signal processing (Arehart et al., 2013). Aging can also be associated with suprathreshold auditory processing changes and alterations of cognitive functions relevant to speech understanding (such as perceptual speed processing, attention, and working memory). These age-related changes may influence several abilities, such as the use of temporal and spectral speech cues, and may therefore modulate the response to specific hearing-aid signal-processing schemes (Pichora-Fuller & Singh, 2006).

### 3.6. Mechanisms Connecting Hearing Loss and Cognition

Two complementary hypotheses are commonly invoked to explain why hearing loss and cognition are linked. The information-degradation hypothesis proposes that effortful compensation for a poor auditory input reallocates cognitive resources away from higher-level processing, so that memory for degraded speech is poorer than for clear speech and downstream comprehension suffers (Panza et al., 2018). The cognitive-reserve, or sensory-deprivation, account proposes instead that chronic hearing loss reduces cognitively stimulating input to the brain over time, with working memory among the domains affected earliest in age-related hearing loss (Panza et al., 2018). The two mechanisms are not mutually exclusive and may operate over different timescales—acute resource reallocation during listening versus cumulative deprivation across years.

Furthermore, a meta-analysis of 33 studies reported that cognition was significantly poorer in adults with untreated hearing impairment and remained poorer in those with treated impairment than in normal-hearing peers (Taljaard et al., 2016). Importantly, the same synthesis indicated that hearing intervention is associated with improved cognitive performance, supporting the view that at least part of the association is potentially modifiable rather than fixed.

### 3.7. Hearing-Aid Intervention and Cognitive Outcomes

Longitudinal cohort studies indicate that hearing-aid use is associated with a reduced cognitive decline. In a 25-year prospective study of 3777 adults aged 65 years and older, self-reported hearing loss predicted greater cognitive decline, but this excess decline was not observed among those who used hearing aids (Amieva et al., 2015). The long follow-up periods in this cohort argue against simple reverse causation and are consistent with the hypothesis that amplification restores cognitively stimulating auditory input. An interesting analysis is that of the ACHIEVE study, the first large randomized controlled trial of hearing intervention also evaluating the cognitive outcomes. It followed 977 adults aged 70–84 years with untreated hearing loss for three years; although hearing intervention did not slow cognitive decline in the overall sample, it has been reported that hearing rehab reduced the onset of cognitive decline by approximately half in the subgroup at higher risk of decline (older participants with more cardiovascular risk factors), indicating that the cognitive benefit of treating presbycusis may be greatest in those who are most vulnerable (Lin et al., 2023). Furthermore, the CogniAid randomized controlled trial tested whether, for older adults with below-average cognition, a “simple” fitting (linear amplification with output-limiting compression) would benefit hearing and cognition more than a “standard” adaptive-compression fitting (Searchfield et al., 2024). Of 67 participants randomized, 48 completed 12-month assessments; although hearing outcomes improved comparably in both groups, the standard fitting produced the greater improvement in fluid cognition (fluid cognition refers to problem-solving abilities that are relatively independent of prior knowledge). This counterintuitive result suggests that the quality of the restored auditory input, rather than processing simplicity per se, drives cognitive benefit—though the small completed sample warrants caution and replication. Convergent neurophysiological work has documented changes in cortical organization and improvements in cognitive function following hearing-aid use in early-stage hearing loss, pointing to neuroplastic mechanisms (Glick & Sharma, 2020).

### 3.8. Artificial Intelligence and Next-Generation Noise Reduction Systems

If the cognitive cost of listening in noise arises largely from the mismatch between a degraded signal and stored phonological representations, then technologies that deliver a cleaner signal should, in principle, lower that cost. Conventional noise reduction algorithms achieve only modest improvements and, as discussed above, can distort the signal in ways that interact with cognition. The most significant recent advance is the application of deep learning to single-microphone noise reduction. Deep neural networks trained on very large databases of noisy speech can separate speech from competing noise far more effectively than traditional approaches, even in adverse conditions such as multi-talker babble and reverberation (Edwards, 2020).

The clinical potential is substantial. In a controlled evaluation, a deep learning algorithm that selectively suppressed noise while preserving the speech signal restored intelligibility for hearing-aid users toward the level of normal-hearing controls, corresponding to a clinically meaningful improvement in speech-reception threshold (Diehl et al., 2023). Within the framework of this review, the relevance of such gains is not only acoustic but cognitive: by reducing phonological mismatch at the input stage, effective artificial-intelligence-based processing should free explicit working-memory resources that would otherwise be consumed in reconstructing degraded speech, with the largest benefit expected precisely for listeners with limited working-memory capacity. Realizing this in wearable devices remains constrained by the need for very low processing latency and by the limited power and memory of hearing aids, so that algorithmic complexity must be matched to on-device hardware (Diehl et al., 2023). A further prospect is processing that adapts in real time to the listening context and, potentially, to the listener’s cognitive load, tailoring noise reduction and compression dynamically rather than applying fixed settings (Edwards, 2020).

For example, a recent deep learning method for implementing both noise reduction and WDRC, called Neural-WDRC (Wide-dynamic-range compression), has been recently tested and compared to traditional methods like slow- and fast-acting WDRC and with a signal-to-noise ratio (SNR) algorithm. It was shown that Neural-WDRC effectively reduced negative interactions of speech and noise in highly non-stationary noise scenarios. WDRC automatically adjusts the volume of soft and loud sounds, compressing a wide range of acoustic volumes into a smaller, tolerable range. Unfortunately, a too-fast compression can alter the natural rise-and-fall of speech, which may interfere with consonant recognition, especially in reverberant rooms where speech perception and listening comprehension are naturally disrupted in both children and adults with normal hearing (Klatte et al., 2010). For individuals with lower working memory, the continuous gain adjustments of the acoustic signal caused by active WDRC can increase listening effort, making it harder to process speech in adverse conditions. Several AI-based tools could further enhance hearing-aid processing. In our opinion, particularly promising could be (i) the Neural-WDRC and integrated processing, which combine wide-dynamic-range compression with controllable noise reduction using deep learning (Zhang et al., 2025), and (ii) the deep neural network-based speech enhancement and noise management—artificial neural networks with multiple processing stages that can be trained to discriminate speech from background noise and to estimate or reconstruct a cleaner speech signal (Diehl et al., 2023).

Beyond compression and noise reduction, according to some Authors, machine-learning methods are also increasingly embedded in other hearing-aid features, including automatic acoustic-scene classification and AI-driven adaptive directionality, which adjust processing to the listening environment in real time (Edwards, 2020).

The present review focuses on hearing aids; however, cochlear implants (CIs) represent another important tool to restore hearing in severe to profound hearing loss. CI systems integrate several signal-processing methods derived from hearing aids but adapt them to the specific requirements of electric hearing, and speech understanding with a CI likewise depends on cognitive resources such as working memory, particularly in noise (Wouters et al., 2015). Extending the present framework to CI signal processing represents a natural direction for future efforts.

## 4. Discussion

Two practical features may be considered from the evidence analyzed. First, because cognitive abilities contribute independently to speech-in-noise outcomes, audiological assessment may benefit from incorporating brief cognitive and speech-in-noise screening, both to identify targets for rehabilitation and to calibrate expectations about aided speech understanding (Mamo et al., 2019). Second, because the cognitive demands of amplification depend on the processing strategy, fitting might be individualized to the listener’s cognitive profile rather than prescribed solely from the audiogram by reconsidering aggressive fast-acting compression for listeners with limited working-memory capacity. Realizing these aims requires interprofessional collaboration in which hearing status informs cognitive care and cognitive status informs hearing care (Panza et al., 2018). This logic applies across the lifespan. Because central auditory processing difficulties can occur despite normal peripheral hearing and have identifiable neurobiological substrates, an assessment that distinguishes auditory and cognitive contributions is valuable in both children and older adults (Bianchino et al., 2026). In practical terms, the audiological work-up could incorporate brief, validated cognitive measures—such as reading-span-type tasks or simple processing-speed tests—particularly for patients with greater hearing loss (Mamo et al., 2019). Beyond identifying rehabilitation targets, such information supports realistic counselling: when a listener is advised that benefit from amplification depends partly on cognitive capacity and not on device sophistication alone, expectations can be calibrated and disappointment reduced. This is especially relevant in presbycusis, where reduced working-memory capacity and processing-induced distortion compound one another (Arehart et al., 2013).

Fitting itself could be arranged by including cognitive tests rather than being audiogram-driven. For listeners with limited working memory, slower compression time constants and gentler noise reduction may lower the cognitive cost of signal distortion, whereas listeners with greater capacity may tolerate, or benefit from, a deeper processing (Souza et al., 2015; Arehart et al., 2013). The demonstration that two fitting strategies producing comparable audibility can yield different speech perception in noise outcomes highlights the fact that signal-processing choices are not cognitively neutral and should be individualized (Searchfield et al., 2024).

Finally, the device is only one element of rehabilitation. Auditory training and combined cognitive–auditory programs (for example, computerized auditory training such as Listening and Communication Enhancement, LACE, or clinician-guided auditory–cognitive training) may help listeners adapt to complex processing over time, and consistent hearing-aid use has been associated with neuroplastic reorganization and gains in cognitive function in early-stage hearing loss (Glick & Sharma, 2020). Since timely hearing intervention is likely to slow cognitive decline in older adults at higher risk (Lin et al., 2023), early detection and treatment of presbycusis represents a public-health opportunity as much as an individual clinical one.

Several priorities follow these considerations. Longitudinal study designs with extended follow-up are necessary to determine how listeners—especially those with smaller working-memory capacity—adapt to complex processing through perceptual learning, since short-term laboratory studies may underestimate eventual benefit (Souza et al., 2015). Fitting research should move toward cognitively individualized prescriptions, matching compression time constants and noise reduction strength to processing speed and working-memory capacity rather than to the audiogram alone (Mamo et al., 2019). Combining cognitive training with auditory rehabilitation may clarify whether targeted intervention can enhance hearing-aid benefit and support neuroplastic reorganization (Glick & Sharma, 2020). Finally, advances in machine-learning-based signal processing may eventually enable amplification that adapts in real time to listening context and, potentially, to cognitive load, reducing the processing burden imposed on listeners with limited cognitive resources (Edwards, 2020).

A major limitation of this manuscript is its narrative nature, as a formal assessment of the methodological quality of the included studies was not performed, even if priority was given to studies with clear methodological descriptions and direct relevance to analyzed topics.

## 5. Conclusions

Speech perception in noise is a coordinated neurocognitive process rather than a purely auditory task. Working memory is a central cognitive contributor, with effects that become evident specifically under adverse conditions and most pronounced when fast-acting compression substantially alters the signal. According to the recent literature, on a side, appropriately fitted hearing aids (i.e., devices verified to prescriptive targets, for example by real-ear measurements) can enhance not only communication but also cognition itself; on the other side, the choice of signal-processing strategy may influence cognitive load, and therefore also speech understanding, particularly for listeners with working-memory or perceptual speed processing limitations.

It is likely that, in the near future, artificial intelligence and deep learning strategies could play a major role in adapting hearing aids to cognitive resources, personalizing the amplification parameters.

## Figures and Tables

**Table 1:** The principal studies informing the review.

Study	Design and Sample	Main Finding Relevant to This Review
Foo et al. (2007)	Experimental; 32 experienced hearing-instrument users	Reading-span working memory explained a large share of variance (~40%) in speech-reception thresholds with unfamiliar fast-compression settings.
Souza et al. (2015)	Narrative review of signal-processing and cognition studies	Working memory matters for fast- but not slow-acting compression; noise reduction interactions are conditional on capacity and algorithm.
Amieva et al. (2015)	Prospective cohort; 3777 adults ≥ 65 y; 25-year follow-up	Excess cognitive decline associated with hearing loss was not observed in hearing-aid users.
Taljaard et al. (2016)	Meta-analysis; 33 studies	Cognition poorer in untreated and treated hearing loss vs. normal hearing; hearing intervention improves cognition.
Yumba (2017)	Cross-sectional; 194 hearing-aid users, 33–80 y, mild–moderate SNHL	Cognitive processing speed predicted aided speech-in-noise better than working memory, regardless of signal-processing algorithm.
Mamo et al. (2019)	Cross-sectional; 250 older adults (mean 77 y), ARIC	Each 1-dB increase in SNR loss linked to lower scores across cognitive domains; memory most robustly associated.
Searchfield et al. (2024)	RCT (CogniAid); 67 randomised, 48 completed 12 mo	Standard adaptive-compression fitting improved fluid cognition more than a simple fitting, despite comparable hearing gains.
Bugannim et al. (2025)	Lifespan cross-sectional; 357 individuals, 7–90 y	Hearing sensitivity declined from ~17 y, cognition from ~32–38 y; cognitive and auditory factors explained ~61% of variance in speech-in-noise.

**Table 2:** Hearing-aid signal-processing strategies and their interaction with cognition.

Processing Strategy	Effect on the Acoustic Signal	Interaction with Cognition
Linear amplification	Increases audibility with minimal alteration of the temporal envelope	Low explicit working-memory demand; serves as a low-distortion reference condition (Foo et al., 2007).
Slow-acting WDRC	Gradual gain changes that largely preserve the amplitude envelope	Outcomes driven mainly by age and degree of hearing loss; largely independent of working-memory capacity (Foo et al., 2007)
Fast-acting WDRC	Smooths the amplitude envelope through rapid gain changes	Greater explicit working-memory demand; benefit depends on working-memory capacity, especially with unfamiliar settings (Souza et al., 2015; Foo et al., 2007)
Digital noise reduction	Suppresses noise but may inadvertently remove speech components	Conditional interactions; larger capacity can aid recall, yet may show disbenefit from strong (not moderate) noise reduction (Foo et al., 2007; Yumba, 2017)

## Data Availability

No new data were created or analyzed in this study. Data sharing is not applicable to this article.
